# Association of physical activity and socio-economic status on mortality in older adults: a retrospective cohort study of KNHANES-mortality linked data

**DOI:** 10.1038/s41598-024-62216-7

**Published:** 2024-06-24

**Authors:** Soomin Lee, Xiaonan Ma, Younghwan Choi, Yeon Soo Kim

**Affiliations:** 1 Department of Physical Education, College of Education, Seoul National University, 1 Gwanak-ro, Gwanak-gu, Seoul, 08826 Republic of Korea; 2 Institute of Sport Science, Seoul National University, Seoul, Republic of Korea

**Keywords:** Lifestyle modification, Geriatrics, Epidemiology

## Abstract

We examined the joint association of physical activity (PA) and socio-economic status (SES) on all-causes and cardiovascular disease (CVD) mortality in 6945 elderly Koreans (mean age: 71.6 years, 41.8% male) using data from the Korean National Health and Nutrition Examination Survey (2007–2013) and death data from Statistics Korea (2019). The SES included household income and education level. PA was assessed using the IPAQ and categorized according to the 2018 PA Guidelines. In stratified analyses using Cox proportional hazards by SES adherence to PA guidelines those who low household income group was associated with a reduced risk of all-cause mortality and CVD mortality, while in the lowest educational level group, it was associated with a reduced risk of all-cause mortality and CVD mortality. In the joint analysis, PA was associated with a significant reduction in all-cause mortality in all groups when compared with those who did not meet PA those who had the lowest SES. However, PA with CVD mortality risk was not significantly associated in the ‘upper-middle’ income and ‘high school’ education groups. The study revealed that PA significantly association mortality, particularly among older adults with low SES. This finding suggests the potential for targeted government interventions to promote healthy aging.

## Introduction

Socioeconomic status (SES), a concept that encompasses economic status, education, occupational status, is closely related to an individual's health. The issue continues to be a matter of global concern^1,2^. As South Korea becomes the super-aged society in the world, health inequalities among older adults are increasing^3,4^. In particular, health inequalities are high due to differences in income group and education levels^5,6^. Understanding SES is essential in addressing health inequalities in older adults and providing preventive care. Despite the similar health status, all-cause mortality is higher for older adults in low-income group^7,8^. In contrast, those with higher education levels and more prestigious jobs tend to live longer than those without these amenities^9^. Similarly, European countries found that mortality and self-rated health were significantly higher in lower SES groups^10^. Studies have shown that persistently low-income group can contribute to the presence of cardiovascular risk factors and delayed CVD treatment, which can increase mortality. In addition, low levels of education increase both the number of CVD risk factors and the likelihood of death from CVD It is thus necessary to understand the antecedents affecting an individual's ability to maintain and improve their health, even at low SES levels^10^.

One strategy to reduce the risk of mortality in older adults is to modify unhealthy behaviors. However, compared to curbing other addictive behaviors such as smoking or drinking, PA is relatively easy to initiate, making it an attractive option for encouraging healthy behavior change^11,12^. Many studies have already shown a positive relationship between older adults and PA. It is worth noting that high PA levels are associated with a significantly lower risk of all-cause mortality or premature death in older adults^13^. Daily brisk walking is also associated with a reduced risk of CVD mortality^14^. Participation in PA in older adults is associated with experiencing healthy aging 10 years later^15^. For older adults, PA is known to play a large role in disease prevention^16^. However, in South Korea, only 16% of adults meet PA guidelines, and only 7% of older adults are physically active^17^. Lifestyle factors such as smoking, physical inactivity, and alcohol consumption account for the largest proportion of premature deaths among older adults^18^. Previous studies have found little evidence of an association between PA and mortality risk in older adults after accounting for SES, and most have examined lifestyle as a whole, making it unclear what adherence to PA guidelines practice alone has^18–20^. Rather than trying to change the SES of older adults, lifestyle modifications can be a feasible approach to healthy aging. Therefore, identifying the risk of mortality by PA levels at specific socioeconomic levels can provide an evidence base for promoting healthy longevity in all SES groups. This study will can help mitigate health inequalities and give everyone, regardless of SES, the opportunity to pursue a healthy lifestyle. We seek to determine whether engaging in recommended levels of PA can mitigate mortality risks across different socioeconomic groups, thereby providing a feasible strategy for promoting healthier aging. This approach could help bridge health inequalities and offer practical insights into public health strategies aimed at enabling all individuals, regardless of SES, to achieve a healthier lifestyle.

## Results

**Figure 1:**
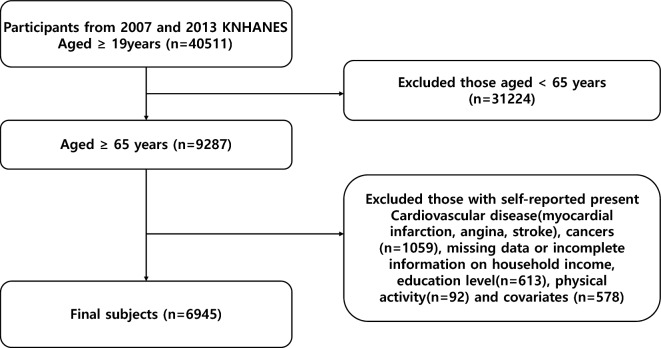
A flow chart showing the number of cases at each stage.

The study was conducted from 2007 to 2013. We initially identified 40,511 participants aged 19 years and older, and excluded those under 65 years of age (n = 9287). We excluded participants who self-reported having current CVD or cancer (n = 1059), those who provided incorrect values for household income group and education (n = 613), and those who provided invalid responses to the PA questionnaire (n = 92). Finally, we excluded participants who missing covariates (n = 578), leaving a total of 6945 participants for analysis (Fig. 1).

The study comprised 6945 participants (41.8% male) in total, with an average age of 71.6 (SD = 4.6) years. During the follow-up period, 1509 all-cause and 332 CVD deaths occurred. Tables 1 and 2 show the baseline characteristics of the participants’ household income and education level respectively. Table 1 summarizes the participants’ characteristics according to household income. Among the participants, 3648 were in (52.5%) low-income group, 1740 (25%) low-middle income group, 888 (12.7%) upper-middle income group, and 669 (9.6%) had high income group. The low-income group were less likely to be married, more likely to be a present smoker, and less likely to meet the recommend PA guideline. The cases of diabetes, dyslipidemia, and hypertension did not vary significantly with a different household income. Table 2 shows the distribution of the study participants according to their education level. 4822 (69.4%) had an education level of elementary school or lower, 782 (11.2%) had completed middle school, 883 (12.7%) had completed high school, and 458 (6.7%) had completed college or received a higher education. Participants with elementary school education or below were likely to be less married. Additionally, 59.6% participants engaged in ≥ 500 METs-min/week of MVPA, and 59.7% had hypertension. Participants with higher income or education group had a higher proportion of meeting the recommended PA guideline.

**Table 1:** Baseline Characteristics according to household income levels.

Characteristics	Overall (n = 6945)	Household income^a^				
		Low (n = 3648)	Lower-middle (n = 1740)	Upper- middle (n = 888)	High (n = 669)	*P*-value
Age (years)	71.60 ± 4.60	72.30 ± 4.60	70.90 ± 4.50	70.70 ± 4.50	71.30 ± 4.80	< 0.001
Sex						< 0.001
Male	2905(41.80%)	1379(32.70%)	819(47.10%)	413(46.50%)	294(43.90%)	
Female	4040(58.20%)	2269(62.20%)	921(52.90%)	475(53.50%)	375(56.10%)	
Body mass index (kg/m^2^)	23.70 ± 3.19	23.60 ± 3.3	23.90 ± 3.20	23.90 ± 3.10	23.90 ± 2.90	0.022
Marital status, n (%)						< 0.001
Unmarried	23(0.03%)	20(0.50%)	2(0.10%)	1(0.10%)	2(0.30%)	
Widowed or divorced	2317(33.40%)	1357(37.20%)	489(28.10%)	250(28.20%)	221(33.00%)	
Married	4603(66.30%)	2271(62.30%)	1249(71.80%)	637(71.70%)	446(66.70%)	
Smoking, n (%)						< 0.001
Non-smoker	25(0.040%)	2207(60.50%)	1009(58.00%)	524(59.00%)	403(60.20%)	
Former smoker	2317(33.40%)	903(24.80%)	516(29.70%)	253(28.05%)	194(29.00%)	
Present smoker	4603(66.30%)	538(14.70%)	215(12.40%)	111(12.50%)	72(10.80%)	
Alcohol consumption^b^, n (%)						0.886
No heavy drinker	6672(96.10%)	3511(96.20%)	1669(95.90%)	851(95.80%)	641(95.80%)	
Heavy drinker	273(3.90%)	137(3.80%)	71(4.10%)	37(4.20%)	28(4.20%)	
Educational level, n (%)						< 0.001
≤ Elementary school	4822(69.40%)	2944 (80.70%)	1088(62.50%)	470(52.90%)	320(47.80%)	
Middle school	782(11.30%)	327(9.00%)	266(15.30%)	110(12.40%)	79(11.80%)	
High school	883(12.70%)	301(8.30%)	268(15.40%)	183(20.60%)	131(19.60%)	
≥ College	458(6.60%)	76(2.10%)	118(6.80%)	125(14.10%)	139(20.80%)	
Total volume of MVPA^c^						< 0.001
< 500 METs-min/week	2497(36.00%)	1422(39.00%)	606(34.80%)	274(30.90%)	195(29.10%)	
≥ 500 METs-min/week	4448(64.00%)	2226(61.00%)	1134(65.20%)	614(69.10%)	474(70.90%)	
Medical history						
Diabetes	1404(20.20%)	732(20.10%)	335(19.30%)	189(21.30%)	148(22.10%)	0.346
Dyslipidemia	3341(48.10%)	1716(47.00%)	858(49.30%)	443(49.90%)	324(48.40%)	0.280
Hypertension	4085(58.80%)	2161(59.20%)	1000(57.50%)	511(57.50%)	413(61.70%)	0.211

Baseline characteristics of according to household income levels. Values are numbers(percentage) unless otherwise noted.

^a^Household income was calculated using equivalised income (total household income/square root of the number of household members).

^b^Alcohol consumption, participants were categorized into non-heavy drinkers and heavy drinkers (defined as at ≥ 7 drinks at least twice a week for male and ≥ 5 drinks at least twice a week for female).

^c^Total volume of moderate to vigorous physical activity was classified according to the 2018 PA Guidelines for Americans. *MVPA* moderate to vigorous physical activity, *METs* metabolic equivalents.

**Table 2:** Baseline Characteristics according to education levels.

Characteristics	Overall (n = 6945)	Education				
		≤ Elementary school (n = 4822)	Middle school (n = 782)	High school (n = 883)	≥ college (n = 458)	*P*-value
Age (years)	71.60 ± 4.60	72.10 ± 4.70	70.50 ± 4.20	70.60 ± 4.20	70.80 ± 4.20	< 0.001
Sex						< 0.001
Male	2905(41.8%)	1417(29.4%)	499(63.8%)	611(69.2%)	378(82.5%)	
Female	4040(58.2%)	3405(70.6%)	283(36.2%)	272(30.8%)	80(17.5%)	
Body mass index (kg/m^2^)	23.70 ± 3.19	23.80 ± 3.30	23.80 ± 2.90	23.70 ± 3.00	23.40 ± 2.80	0.158
Marital status, n (%)						< 0.001
Unmarried	23(0.03%)	16(0.30%)	5(0.60%)	2(0.20%)	2(0.40%)	
Widowed or divorced	2317(33.40%)	2009(41.70%)	135(17.30%)	128(14.50%)	45(9.80%)	
Married	4603(66.30%)	2797(58.00%)	642(82.10%)	753(85.30%)	411(89.70%)	
Smoking, n (%)						< 0.001
Non-smoker	25(0.04%)	3241(67.20%)	359(45.90%)	384(43.50%)	159(34.70%)	
Former smoker	2317(33.40%)	979(20.30%)	291(37.20%)	347(39.30%)	249(54.40%)	
Present smoker	4603(66.30%)	602(12.50%)	132(16.90%)	152(17.20%)	50(10.90%)	
Alcohol consumption^a^, n (%)						< 0.001
No heavy drinker	6672(96.10%)	4662(96.70%)	740(94.60%)	832(94.20%)	438(95.60%)	
Heavy drinker	273(3.90%)	160(3.30%)	42(5.40%)	51(5.80%)	20(4.40%)	
Household income^b^, n (%)						< 0.001
Low	4822(69.40%)	2944(61.60%)	327(41.80%)	301(34.10%)	76(16.60%)	
Lower-middle	782(11.30%)	1088(22.60%)	266(34.00%)	268(30.40%)	118(25.80%)	
Upper-middle	883(12.70%)	470(9.70%)	110(14.10%)	183(20.70%)	125(27.30%)	
High	458(6.60%)	320(6.60%)	79(10.10%)	131(14.80%)	139(30.30%)	
Total volume of MVPA^c^						< 0.001
< 500 METs-min/week	2497(36.00%)	1949(40.40%)	242(30.90%)	223(25.30%)	83(18.10%)	
≥ 500 METs-min/week	4448(64.00%)	2873(59.60%)	540(69.10%)	660(74.70%)	375(81.90%)	
Medical history						
Diabetes	1404(20.20%)	958(19.90%)	177(22.60%)	175(19.80%)	94(20.50%)	0.346
Dyslipidemia	3341(48.10%)	2299(47.70%)	393(50.30%)	436(49.40%)	213(46.50%)	0.416
Hypertension	4085(58.80%)	2878(59.70%)	433(55.40%)	524(59.30%)	250(54.60%)	0.032

Baseline characteristics of according to household education levels. Values are numbers(percentage) unless otherwise noted.

^a^Alcohol consumption, participants were categorized into non-heavy drinkers and heavy drinkers (defined as at ≥ 7 drinks at least twice a week for male and ≥ 5 drinks at least twice a week for female).

^b^Household income was calculated using equivalised income (total household income/square root of the number of household members).

^c^Total volume of moderate to vigorous physical activity was classified according to the 2018 PA Guidelines for Americans. *MVPA* moderate to vigorous physical activity, *METs* metabolic equivalents.

Table 3 illustrates the link between SES (household income and education level) and PA with all-cause and CVD mortality, respectively. The risks of all-cause and CVD mortality in the high-income group were significantly lower than those in the low-income group (hazard ratio [HR] 0.80, 95% confidence interval [95% CI] 0.66–0.98 in all-cause mortality; HR 0.47, 95% CI 0.28–0.77 in CVD mortality). Regarding education level, individuals with a college or higher education level had significant association with all-cause mortality than those with elementary school education level or below (HR 0.62, 95% CI 0.48–0.80). However, education was not significantly associated with CVD mortality (HR 0.65, 95% CI 0.37–1.15). Finally, we examined the relationship between PA and all-cause and CVD mortality. The group with 500 METs-min/week or more demonstrated a significant association with decreased risk of all-cause and CVD mortality (HR 0.84, 95% CI 0.76–0.94; HR 0.73, 95% CI 0.59–0.90) compared to the group with 500 METs-min/week or less. All variables were mutually adjusted for covariates in each analysis: age, sex, BMI, marital status, alcohol consumption, education, household income, smoking, diabetes, dyslipidemia, and hypertension.

**Table 3:** Rates and hazard ratio for all-cause and cardiovascular disease mortality according to household income, education and physical activity level.

	All-cause mortality			CVD mortality	
	Total participants (n = 6945)	No. of deaths (n = 1509)	Adjusted HR (95% CI)	No. of deaths (n = 332)	Adjusted HR (95% CI)
Household income					
Low	3648	904	1(Reference)	228	1(Reference)
Lower-middle	1741	329	0.95(0.84–1.08)	74	0.88(0.67–1.15)
Upper-middle	888	162	0.96(0.80–1.13)	43	1.03(0.74–1.44)
High	669	114	0.80(0.66–0.98)	17	0.47(0.28–0.77)
Education					
≤ Elementary school	4823	1105	1(Reference)	281	1(Reference)
Middle school	782	159	0.90(0.76–1.08)	32	0.86(0.59–1.25)
High school	883	173	0.86(0.72–1.02)	35	0.83(0.57–1.21)
≥ College	458	72	0.62(0.48–0.80)	14	0.65(0.37–1.15)
Total volume of MVPA					
< 500 METs-min/week	2497	614	1(Reference)	168	1(Reference)
≥ 500 METs-min/week	4449	895	0.84(0.76–0.94)	194	0.73(0.59–0.90)

Household income, education and total volume of MVPA were mutually adjusted as exposures in different analyses. *CVD* cardiovascular disease, *HR* hazard ratio, MVPA moderate to vigorous physical activity, *METs* metabolic equivalents.

Table 4 shows the results of the association of PA with all-cause and CVD mortality stratified by SES. The association of PA with all-cause mortality was modified by SES. Significant association was observed in the low-income group (HR 0.78, 95% CI 0.68–0.89). No significant association was found in other household income levels. For education level and risk of all-cause mortality, significant association was observed for elementary school or less (HR 0.87, 95% CI 0.77–0.98) and for middle school (HR 0.67, 95% CI 0.48–0.94). We also examined the association with PA with CVD mortality risk stratified by SES. Significant associations were identified with CVD mortality risk by household income level (HR 0.72, 95% CI 0.55–0.94 for low-income group and HR 0.58, 95% CI 0.36–0.94 for lower-middle income group). Educational level was also significantly associated with CVD mortality risk (HR 0.72, 95% CI 0.60–0.96 for elementary school or less, HR 0.47, 95% CI 0.22–0.99 for middle school and HR 0.28, 95% CI 0.09–0.92 for college or higher).

**Table 4:** Hazard ratios for all-cause mortality and cardiovascular diseases mortality according to physical activity stratified by social-economic status.

	Total volume of MVPA	All-cause mortality		CVD mortality	
		Events	Adjusted HR (95% CI)	Events	Adjusted HR (95% CI)
Household income					
Low	< 500 METs-min/week	398	1.00(Reference)	110	1.00(Reference)
	≥ 500 METs-min/week	506	0.78(0.68–0.89)	118	0.72(0.55–0.94)
Lower-middle	< 500 METs-min/week	115	1.00(Reference)	38	1.00(Reference)
	≥ 500 METs-min/week	214	1.04(0.82–1.31)	36	0.58(0.36–0.94)
Upper-middle	< 500 METs-min/week	64	1.00(Reference)	15	1.00(Reference)
	≥ 500 METs-min/week	98	0.73(0.52–1.02)	28	0.03(0.42–1.61)
High	< 500 METs-min/week	37	1.00(Reference)	5	1.00(Reference)
	≥ 500 METs-min/week	77	1.26(0.83–1.91)	12	1.63(0.54–4.93)
Education					
≤ Elementary school	< 500 METs-min/week	491	1.00(Reference)	139	1.00(Reference)
	≥ 500 METs-min/week	614	0.87(0.77–0.98)	142	0.76(0.60–0.96)
Middle school	< 500 METs-min/week	55	1.00(Reference)	13	1.00(Reference)
	≥ 500 METs-min/week	104	0.67(0.48–0.94)	19	0.47(0.22–0.99)
High school	< 500 METs-min/week	47	1.00(Reference)	10	1.00(Reference)
	≥ 500 METs-min/week	126	0.95(0.68–1.35)	25	0.94(0.44–2.00)
≥ College	< 500 METs-min/week	21	1.00(Reference)	6	1.00(Reference)
	≥ 500 METs-min/week	51	0.61(0.36–1.04)	8	0.28(0.09–0.92)

Models was adjusted for age, sex, body mass index(kg/m^2^), marital status (unmarried/widowed or divorced/married), education (≤ elementary school/middle school/high school/ ≥ college), alcohol consumption (non-heavy drinker/heavy drinker), smoking status (non-smoker/former smoker/current), diabetes (yes/no), dyslipidemia (yes/no) and hypertension (yes/no). *p < 0.001 *CVD* cardiovascular disease, *HR* hazard ratio, *MVPA* moderate to vigorous physical activity, *METs* metabolic equivalents.

**Figure 2:**
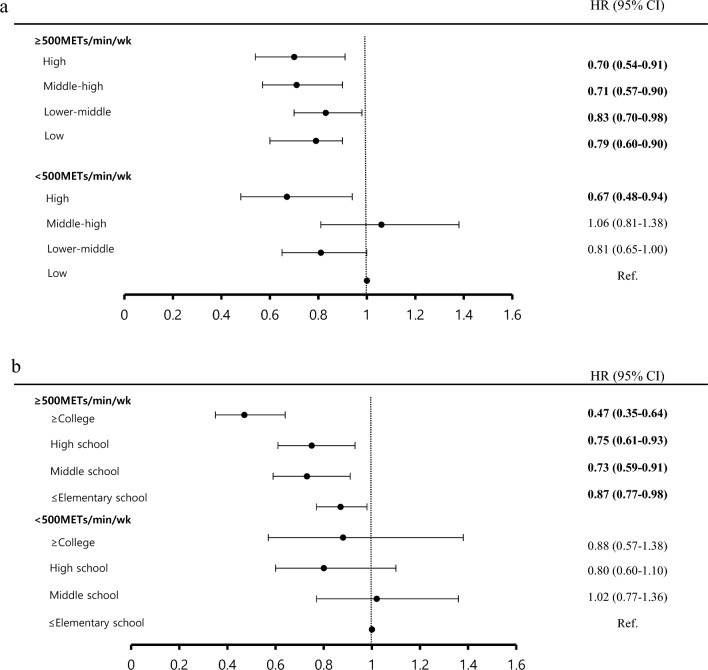
Joint analysis of socio-economic status and physical activity level with all-cause mortality. Joint analysis of household income and physical activity level with all-cause mortality (**a**). Models was adjusted for age, sex, body mass index(kg/m^2^), marital status (unmarried/widowed or divorced/married), education (≤ elementary school/middle school/high school/ ≥ college), alcohol consumption (non-heavy drinker/heavy drinker), smoking status (non-smoker/former smoker/current), diabetes (yes/no), dyslipidemia (yes/no) and hypertension (yes/no). *p < 0.001 *METs* metabolic equivalents. Joint analysis of education level and physical activity level with all-cause mortality (**b**). Models was adjusted for age, sex, body mass index(kg/m^2^), marital status (unmarried/widowed or divorced/married), household income (low/lower-middle/middle-high/high), alcohol consumption (non-heavy drinker/heavy drinker), smoking status (non-smoker/former smoker/current), diabetes (yes/no), dyslipidemia (yes/no) and hypertension (yes/no). *p < 0.001 *METs* metabolic equivalents.

The joint association of SES and PA levels with all-cause mortality is shown in Fig. 2. Figure 2a shows that the risk of all-cause mortality in all household income groups was significantly lower than in the low-income group for those who did not adhere to PA (HR 0.70, 95% CI 0.54–0.91 in the high-income group, HR 0.71, 95% CI 0.57–0.90 in the middle-high income group, HR 0.83, 95% CI 0.70–0.98 in the low-middle income group, and HR 0.79, 95% CI 0.69–0.90 in the lowest income group). Finally, we identified the association among education level, PA, and all-cause mortality, which is depicted in Fig. 2b. The risk of all-cause mortality in met the PA those with all education levels were significantly lower than in the elementary school or lower group for those who did not adherence PA (HR 0.47, 95% CI 0.35–0.64 in the above college group; HR 0.75, 95% CI 0.61–0.93 in the high school group; HR 0.73, 95% CI 0.59–0.91 in the middle school; HR 0.87, 95% CI 0.77–0.98 in the elementary school or lower). Figure 2a and b present the result adjusted for covariates such as age, sex, BMI, marital status, alcohol consumption, smoking, diabetes, dyslipidemia, and hypertension. Figure 2a was further adjusted for education level and Fig. 2b for household income.

**Figure 3:**
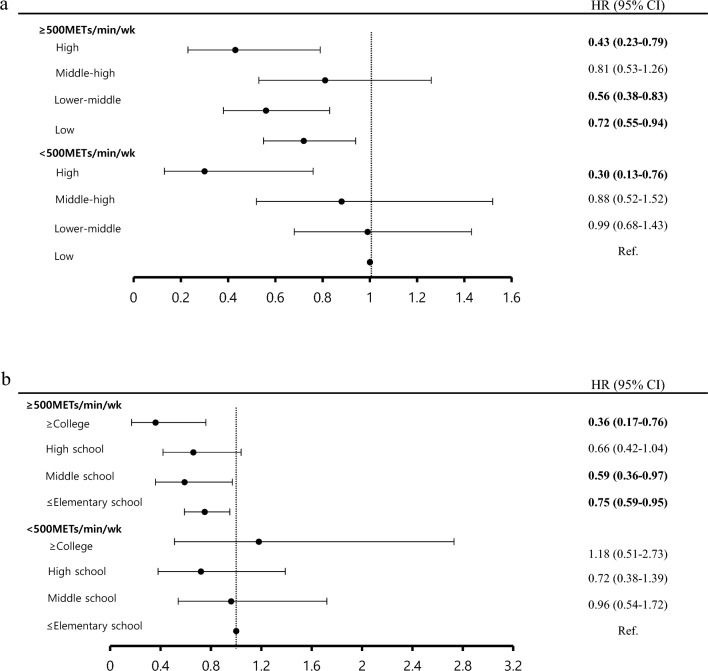
Joint analysis of socio-economic status and physical activity level with cardiovascular diseases mortality. Joint analysis of household income and physical activity level with cardiovascular diseases mortality (**a**). Models was adjusted for age, sex, body mass index(kg/m^2^), marital status (unmarried/widowed or divorced/married), education (≤ elementary school/middle school/high school/≥ college), alcohol consumption (non-heavy drinker/heavy drinker), smoking status (non-smoker/former smoker/current), diabetes (yes/no), dyslipidemia (yes/no) and hypertension (yes/no). *p < 0.001 *METs* metabolic equivalents. Joint analysis of education level and physical activity level with cardiovascular diseases mortality (**b**). Models was adjusted for age, sex, body mass index(kg/m^2^), marital status (unmarried/widowed or divorced/married), household income (low/lower-middle/middle-high/high), alcohol consumption (non-heavy drinker/heavy drinker), smoking status (non-smoker/former smoker/current), diabetes (yes/no), dyslipidemia (yes/no) and hypertension (yes/no). *p < 0.001 *METs* metabolic equivalents.

Figure 3 illustrates the association of CVD mortality with SES and PA.g Figure 3a indicates that the risk of CVD mortality in the all household income groups for those who above 500 METs-min/week of PA (except the middle-high group) was significantly lower than low-income group for those who not met the PA. (HR 0.43, 95% CI 0.23–0.79 in the high-income group; HR 0.81, 95% CI 0.53–1.26 in the middle-high income group; HR 0.56, 95% CI 0.38–0.83 in the low-middle income group; HR 0.72, in the low-income group 95% CI 0.55–0.94). In addition, high-income group engaged in less than 500 METs-min/week had a CVD mortality risk of 0.30 (95% CI 0.13–0.76). Figure 3b shows how the education level and MVPA is related to CVD mortality. The risk of CVD mortality was significantly lower for participants of all education levels (except for the high school graduates) when engaged in more than 500 METs-min/week of MVPA, in comparison to those with an education level lower than elementary school and engaged in less than 500 METs-min/week. Specifically, the group with a college education or higher had a CVD mortality of 0.36 (95% CI 0.17–0.76). For the middle school and elementary school or below group, CVD mortality was 0.59 (95% CI 0.36–0.97) and 0.75 (95% CI 0.59–0.95) respectively. Figure 3a and b indicate the analyses that were controlled for covariates such as age, sex, BMI, marital status, alcohol consumption, smoking, diabetes, dyslipidemia, and hypertension. Figure 3a was further adjusted for education level and Fig. 3b for household income group.

## Discussion

Our findings showed that among people aged 65 years and older, PA of 500 METs-min/week or more was associated with a reduced risk of all-cause and CVD mortality in the low household income and education groups. Similarly, in joint analyses, those who met the PA guidelines had a significantly lower risk of all-cause mortality in all groups (including household income and education level) compared with the lowest SES and who did not meet the PA guidelines. However, for CVD mortality, no significant association were found for 'middle-high’ group and 'high school' for education level.

The link between SES disparity and mortality is already a topic of global interest. A study examining the association between SES levels and mortality risk in older adults in the United States found that higher SES was associated with a reduction in mortality^21^. A large cohort study in the US found similar results, showing that higher income was associated with longer life expectancy^22^. Consistent with previous research, our study also found that higher household income group was independent predicator of risk of all-cause and CVD mortality. The link between household income group and mortality has been well established in previous studies^22,23^. A stable income affords individuals greater opportunities to prioritize their health. This includes making informed dietary choices, striving for a clean and healthy environment, engaging in regular exercise, and effectively managing stress.

Despite previous studies suggesting an association between educational attainment and CVD mortality, the results of this study did not reach statistical significance^8,9^. Although many previous studies have shown similar association, results may vary due to differences in data structure, such as discrepancies in follow-up periods. We believe that more research data are necessary to obtain accurate results in Korean participants.

A higher education level is associated with better health literacy^9^. Differences in education level can lead to differences in health-related knowledge, potentially affecting healthy aging. The absence of substantial findings in this study may be attributed to the limited number of participants, which made it challenging to ascertain the extent of the risk. A larger sample size is necessary to validate the results. Individuals with elementary school or below were found to have a significantly higher risk of all-cause mortality, and were more likely to be obese or have non-communicable diseases. Education can provide basic understanding on the healthy behavior including personal hygiene, regular exercise, and preventive vaccinations. It also provides an opportunity to gain knowledge about healthy behaviors, and help them accurately communicate their health needs to their caregivers or doctors. Education has direct and indirect consequences on an individual's health.

A systematic review of the health status of individuals according to socioeconomic inequalities found that health behaviors vary by socioeconomic level. Lower SES groups have a higher risk of all-cause and CVD mortality through behaviors such as smoking, alcohol consumption and physical inactivity^24^. Recent studies have provided evidence that pursuing a healthy lifestyle is associated with a reduced risk of mortality and cardiovascular risk, even for those with a fixed SES^18,20,25^. However, the findings are contradicted by the diversity of health behaviors associated with mortality risk and the wide age range of the study population. There is a need to understand the health behaviors and outcomes of mortality and chronic disease prevalence that are appropriate for older adults in aging society.

Among the health behaviors that should be modified for healthy aging, PA has an established evidence base for preventing the prevalence of disease in older adults. The association between PA and successful aging in older adults has been proven in many studies. A 15-year follow-up cohort study in Norway explored the association of PA with the risk of all-cause and CVD mortality. It deduced that older adults who engaged in PA had a significantly reduced risk of all-cause and CVD mortality, thereby identifying PA as a protective factor against premature death^13^. Individuals who met the adherence for PA engage reportedly have a reduced risk of heart failure^26^. The results of the present study are aligned with prior research, providing strong evidence that the risk of CVD morbidity and mortality in older adults with non-communicable diseases can be lowered by increasing PA. However, there is still a lack of evidence on the association of PA with all-cause and CVD mortality risk by SES^20,27^. While there are many factors that contribute to unequal mortality risk in older adults, it is important to examine the impact of PA, a health behavior that is easily modifiable due to fixed SES, on mortality risk. In our study, stratified analyses showed a protective effect of PA on all-cause and CVD mortality risk reduction in the low-income group and low education. PA is a relatively easier intervention to implement than smoking and alcohol consumption. Therefore, it is important to emphasize the health benefits of PA. Public health should focus on SES rather than focusing on a cross-sectional view of an individual's health status.

In our study, a significant association was observed between high-income and decreased risk of CVD mortality among individuals not actively engaged in PA. This may be due to the fact that individuals with high household incomes typically have easier access to healthcare services, which makes them more likely to undergo regular health check-ups. Income disparity has a significant impact on various lifestyle aspects. Future research should explore the link between high-income groups and mortality risk, even if they do not engage in PA, and examine the possible underlying factors.

The objective of this study was to examine the association between PA and SES and mortality risk in Asian adults aged 65 years and older. This study has significant social relevance and provides an interdisciplinary perspective on this issue. The findings of this study suggest that older adults with low SES have a reduced risk of mortality when adhering to PA guidelines. The findings of this retrospective cohort study offer insights that can inform the development of national policy. A comprehensive approach can provide a scientific rationale for emphasizing the importance of physical activity in reducing the risk factors associated with health behaviors among older adults. This study had limitations that need consideration. First, we used a self-reported questionnaire to assess PA, which may have led to a self-reported bias. Future studies should use objective measures of PA such as accelerometers to confirm the association between PA and mortality. Second, this study only examined at time to death. Therefore, we cannot estimate the risk of death associated with changes in SES and PA. This prevented us from assessing the changes in SES and PA levels up to the time of death, making it challenging to establish a causal relationship between SES and PA. Third, the results of this study may not apply to groups that are unable to participate in independent daily activities, such as older adults with disabilities that cause functional limitations. Therefore, one should use caution when analyzing these results. Finally, we used the Cox regression statistical method to identify mortality rates in this study. However, this statistical method may not fully measure the complexity of competing risk all-cause and CVD mortality.

In conclusion, this study found that household income and education level, which are indicators of SES, were significantly associated with all-cause and CVD mortality in older adults aged 65 years and older, depending on whether PA was met. Specifically, lower household income and blow of educational level were closely linked to lower all-cause and CVD mortality when PA guidelines were followed. Based on these findings, PA was confirmed to be a protective factor that reduced the mortality risk of an individual linked to their SES. We hope that this research will serve as a basis for developing PA policies tailored to the income and education levels of older adults, so that PA can reduce mortality and promote healthy aging. The findings underscore the significance of PA in reducing health disparities among older adults. Although the results were not consistent between the middle-high income level and high school education or lower levels, it is believed that despite differences in SES, PA can help promote health in older adults. It is now well established that smoking cessation, alcohol abstinence, and PA can prevent chronic disease and extend lifespan. It is recommended that public health professionals and communities consider the socioeconomic status of older adults when formulating policy proposals to reduce the impact of disease and promote healthy aging. It is our hope that this study will provide a reliable basis for the formulation of such proposals.

## Methods

### Study population

This study analyzed the data of individuals who participated in the Korea National Health and Nutrition Examination Survey (KNHANES) between 2007 and 2013 and agreed to provide cause-of-death follow-up data until December 31, 2019. Cause-of-death data were collected by Statistics Korea to provide basic information for the establishment of national welfare and healthcare policies^28^. The KNHANES is a national cross-sectional survey conducted by the Korea Disease Control and Prevention Agency (KDCA) to provide data on health behaviors, eating habits, diets, and nutrition^29^. The KNHANES is annually approved by the Korean Institutional Review Board, adhering to guidelines based on laws like the Declaration of Helsinki since 2007. Participants gave informed consent before the study, and the data used in this study were approved by the KDCA (IRB No. 2007-02con-04-p, 2008-04EXP-01-C, 2009-01con-03-2c, 2010-02con-21-c, 2011-02con-06-c, 2012-01exp-01-2c, 2013-07con-03-4c) and the Seoul National University Institutional Review Board (IRB No. 2311/001-005). As shown in the flowchart in Fig. 1, we restricted to participants aged ≥ 65 years and excluded individuals who self-reported CVD (myocardial infarction, angina and stroke) and cancer at baseline. Finally, we excluded missing or invalid variables for household income, education, PA and other covariates.

### Exposure

#### SES

Household income was categorized into ‘low,’ ‘lower-middle,’ ‘upper-middle,’ and ‘high’ income quartiles in KHNANES. For every survey, the participants were instructed to answer the question "average monthly household gross income" numerically; then, the monthly household income was divided by the square root of number of household members to get the average monthly household equalized income. This income data was categorized by sex and quartile, and specific information was provided in the user guide available on the website^30^. Education level was also classified into quartiles as follows: ≤ elementary school, middle school, high school, and ≥ college.

#### PA

PA was used as the moderator variable and was obtained using the Korean version of the International Physical Activity Questionnaire Short Form (IPAQ-SF), which has been reported to have high validity and reliability in Koreans^31,32^. The IPAQ-SF can estimate the total PA using METs values for moderate-intensity PA, vigorous PA, and walking.

MVPA was calculated using the following formula:

The total volume of MVPA METs-min/week = 3.3 × walking minutes × walking 'day' + 4.0 × moderate-intensity activity minutes × moderate days + 8.0 × vigorous activity minutes × vigorous days^33^.

We categorized participants into 2 groups, those who met MVPA (≥ 500 METs-min/week) requirements and those who met did not (< 500 METs-min/week) according to the 2018 Physical Activity Guidelines for Americans, 2nd edition^34^.

##### Examples

For MVPA ≥ 500 METs/min/week, running with a vigorous intensity of 7 METs, 3 days a week for 30 min each session, totals 630 METs/min/week. This exceeds the 500 METs/min/week threshold, indicating a sufficient level of PA.

For MVPA < 500 METs-min/week: leisurely walking, with a light intensity of 2.8METs, 3 days a week for 50 min each session, a total of 420 METs-min/week. This activity falls below the 500 METs-min/week threshold, reflecting a lower level of PA.

#### Covariates

To minimize confounding in our findings, we selected specific covariates based on those used in previous studies analyzing mortality risk among the elderly in Korea. Instead of using the number of comorbidities, we included physician-diagnosed chronic diseases as covariates to ensure the accuracy of results^35,36^. Covariates included age, sex (male or female), BMI (kg/m^2^), smoking status (non-smoker, former smoker, or current smoker), alcohol consumption (Heavy drinker or non-heavy drinker), and marital status (single, Widowed/divorced or married). Alcohol consumption was classified as a categorical variable and the participants categorized as either heavy drinkers or non-heavy drinkers (≥ 7 drinks at least twice a week for males and ≥ 5 drinks at least twice a week for females). We categorized inclusion based on the diagnosis of chronic conditions (yes or no) with high morbidity in older adults, such as hypertension, diabetes, and dyslipidemia^37^.

### Outcomes

#### Mortality

The causes of death were determined according to the International Classification of Diseases, 10th Revision^38^. Outcomes included all-cause and CVD mortality, which were defined as events that occurred after the baseline assessment. Of the 6945 participants, 1509 died of all-cause mortality, and 362 of CVD mortality.

### Statistical analysis

To confirm the baseline characteristics of the participants based on household income Table 1 and education level Table 2, continuous data were reported as mean ± standard deviation (SD), and categorical variables were presented as percentage. To examine the independent association between SES and PA with mortalities were mutually adjusted using age, sex, BMI, marital status, household income, education level, alcohol consumption, smoking, diabetes, dyslipidemia, and hypertension as potential covariates. Cox proportional hazard models were used to estimate the HR and 95% CI for all-cause and CVD mortality. Stratified analyses were performed to assess whether the relationship between adherence to the PA guidelines and mortality varied based on household income levels and education levels. We selected MVPA < 500 METs-min/week as the reference group in each group of household income level (‘low’, ‘lower-middle’, ‘upper-middle’, and ‘high’) and education level (‘ ≤ elementary school’, ‘middle’ ‘school’, ‘high school’, and ‘ ≥ college’). Multiplicative interactions were assessed by comparing the two models, with and without an interaction term, using the log-likelihood ratio test. To assess the joint association of SES and adherence to PA guidelines with mortality, the participants were cross-classified into eight categories based on SES and levels of adherence to the PA guidelines. Combined analysis was also adjusted for potential covariates including age, sex, BMI, marital status, education level, alcohol consumption, smoking, diabetes, dyslipidemia, and hypertension, as shown in Figs. 2a and 3a. Based on Figs. 2a and 3a, 2b and 3b included the household income variable instead of the education level. All statistical analyses were conducted using R (version 4.2.0), and *P*-value < 0.05 was considered statistically significant.

## Data Availability

All data and materials have been made publicly available at the Korea National Health and nutrition Examination website (https://knhanes.kdca.go.kr/knhanes/main.do).
